# POPPY II Cohort Profile – a population-based linked cohort examining the patterns and outcomes of prescription opioid use in NSW, Australia, 2003-2018.

**DOI:** 10.23889/ijpds.v7i3.1966

**Published:** 2022-08-25

**Authors:** Rhiannon Pilkington, Alicia Montgomerie, Angela Gialamas, John Lynch

**Affiliations:** 1 National Drug and Alcohol Research Centre, UNSW Sydney; 2 Centre for Big Data Research in Health, UNSW Sydney, Sydney Australia; 3 School of Population Health, Faculty of Medicine and Health, UNSW Sydney, Sydney Australia; 1 Menzies Centre for Health Policy, Faculty of Medicine and Health, The University of Sydney, Sydney, Australia; 5 School of Public Health, Faculty of Medicine and Health, The University of Sydney, Sydney, Australia; 6 School of Medicine and Public Health, Faculty of Health, The University of Newcastle, Newcastle Australia; 7 Centre de Recherche du Centre Hospitalier de l'Université de Montréal, Université de Montréal; 8 Faculty of Science, Medicine and Health, University of Wollongong, Wollongong. New South Wales Australia

## Objectives

Although opioid prescribing and harms have increased in Australia, there is a lack of population-level evidence about the drivers of long-term opioid use, dependence and other harms. This study aims to profile the POPPY II cohort, with respect to sociodemographic and clinical health characteristics and patterns of opioid initiation.

## Approach

The POPPY II cohort includes adult residents (≥18 years) in NSW who were initiated on prescribed opioids subsidised through Australia’s Pharmaceutical Benefits Scheme for any period between 1st July 2003 and 31st December 2018. The cohort has been linked to nine other datasets containing information on socio-demographic and clinical characteristics, health service use, and adverse outcomes.

## Results

There were 3,569,433 people in the cohort. One in four people were aged ≥65 years at the time of opioid initiation (26.8%) and half were female (52.7%). About 71% resided in a major city. Approximately 6% had evidence of being treated for cancer in the year prior to opioid initiation (5.8%). In the 3 months prior to cohort entry, 27% used an analgesic medicine and 21% used a psychotropic medicine. Less than a third initiated on a strong opioid (22.2%) and the most commonly initiated opioid was paracetamol/codeine (61.3%).

## Conclusion

The POPPY II study is the largest post-marketing surveillance study of prescribed opioids in Australia, and one of the largest studies worldwide. Understanding the characteristics of the cohort will inform future work aimed at generating robust evidence of the long-terms patterns and outcomes of prescribed opioid use in the Australian community.

